# Drought Stress Interacts With Powdery Mildew Infection in Tomato

**DOI:** 10.3389/fpls.2022.845379

**Published:** 2022-03-08

**Authors:** Sri Sunarti, Christos Kissoudis, Yannick Van Der Hoek, Hanneke Van Der Schoot, Richard G. F. Visser, C. Gerard Van Der Linden, Clemens Van De Wiel, Yuling Bai

**Affiliations:** ^1^Plant Breeding, Wageningen University and Research, Wageningen, Netherlands; ^2^Graduate School Experimental Plant Sciences, Wageningen University, Wageningen, Netherlands

**Keywords:** crosstalk, drought stress, ethylene, powdery mildew resistance, tomato

## Abstract

Under field conditions, plants are often exposed to more than one stress factor at the same time, and therefore need to adapt to different combinations of stresses. Crosstalk between responses to abiotic and biotic stresses is known to occur, and the interaction between stress responses can be positive or negative. We studied the interaction of drought stress and powdery mildew (PM) infection in tomatoes using near-isogenic tomato lines (NILs) carrying the *Ol-1, ol-2*, or *Ol-4* gene that confers resistance to tomato PM caused by *Oidium neolycopersici*. Our study demonstrated that drought-induced growth reduction was not further reduced by powdery mildew infection. Drought stress, however, decreased fungal infection in the susceptible genotype Moneymaker (MM) with fungal biomass tending to decrease further as the drought severity increased. Drought stress did not affect PM resistance levels of resistant NIL carrying *ol-2* (a mutant of the tomato susceptibility *Mlo* gene) and *Ol-4* an NLR (nucleotide-binding site-LRR) R gene associated with a fast hypersensitivity response (HR) but tended to slightly decrease disease levels of NIL-Ol-1 (no gene characterized yet, associated with a slow HR following PM infection). At the molecular level, genes involved in abscisic acid (ABA), salicylic acid (SA), and ethylene pathways were highly induced under combined stress indicating the involvement of ABA, SA, and ethylene in the crosstalk between abiotic and biotic stress. Messenger RNA expression of the ABA-responsive dehydrin *SlTAS14* was induced under drought and combined stress with the highest induction under combined stress, and resistant NIL lines showed higher expression levels than MM. The expression of *SlNCED* (involved in ABA synthesis) was also upregulated under drought and highly induced under combined stress. Expression levels of pathogen responsive gene *SlPR1* (an indicator of the SA pathway) and *SlACS* (involved in ethylene synthesis) were highly induced under powdery mildew infection in MM and the *Ol-1* and were induced the most under combined stress in these lines. Taken together, these findings indicate that drought stress can interact with and influence PM infection in tomatoes in a resistance type-dependent manner. The role of hormonal signaling pathways in the crosstalk between drought stress and PM infection is further discussed.

## Introduction

Crop yields worldwide are affected by environmental factors, both biotic and abiotic. Resistance to pathogens, insects, and pests has been studied elaborately and in detail for a long time, and more recently, more and more studies are targeting tolerance to the most important abiotic stresses, such as drought, salinity, or high temperature. Under field conditions, crop plants are often subjected to more than one stress (for instance drought combined with heat, but also diseases combined with salinity or drought), and evidence shows that the response to one stress factor can positively or negatively affect the response to another stress factor. For example, salt stress increased susceptibility to the hemibiotrophic bacterial pathogen *Pseudomonas syringae* pv. tomato (Pto), the necrotrophic fungus *Alternaria brassicicola*, and *Botrytis cinerea* in *Arabidopsis* (Haller et al., 2020). On the other hand, a study on the effects of drought and salt stress on the interaction of tomato (*Solanum lycopersici*) with the biotrophic fungus powdery mildew *Oidium neolycopersici* and the necrotrophic fungus *Botrytis cinerea* reported that drought led to significant suppression of infections by both pathogens, while salt only affected the *Oidium* infection (Achuo et al., 2006). These studies point to the involvement of different mechanisms employed by the plant in response to different combinations of biotic and abiotic stress factors. Indeed, several studies suggest that the response to a combination of stress factors is not merely the sum of the individual stress responses, and therefore cannot be predicted from the responses to single stresses (Ramegowda and Senthil-Kumar, 2015). Therefore, studying the response of plants exposed to combinations of stress factors is essential to gain insight into stress response interactions and to improve crop yields under stressful field conditions (Kissoudis et al., 2014; Bai et al., 2018).

Interactions between responses to different stress factors are evident at the phenotypic level, and many studies indicate that these interactions reside in crosstalk at the physiological and molecular levels. Key elements of the signaling pathways in response to stress include transcription factors (TFs), hormonal pathways, and ROS (reactive oxygen species) and these are suggested to play important roles in the crosstalk between stress response pathways. For example, members of the WRKY family of TFs were shown to be involved in responses to both abiotic and biotic stress factors (Bai et al., 2016). A tomato SlWRKY8 transcription factor was shown to function as a positive regulator for plant resistance to *P. syringae* pv. tomato as well as for drought and salt stress tolerance (Gao et al., 2020). SlWRKY8 is likely to interact with several hormonal pathways: with abscisic acid (ABA) as a pivotal pathway in the drought and salt stress response, and salicylic acid (SA), which is usually associated with defense response to biotrophic pathogens. Regarded as antagonistic to SA, jasmonic acid (JA) is typically associated with responses to necrotrophic pathogens, but there are exceptions. For instance, SA was reported not to be effective against the biotroph *O. neolycopersici* but to have a role in resistance against the necrotrophic *B. cinerea* in tomatoes (Achuo et al., 2004). Besides playing an important role in the senescence of plants and fruits, ethylene (ETH) signaling can be involved in both abiotic and biotic stresses. Transcriptome analysis showed the response of wild *Arachis* exposed to simultaneous drought and nematodes mainly appeared to involve an ETH signaling pathway, whereas drought alone most obviously appeared to affect an ABA signaling pathway and nematode infection a JA signaling pathway. This demonstrates that the response to the combined abiotic and biotic stress was distinct from that to the individual stresses. Likewise, ROS is involved in both biotic and abiotic stress responses including (hypersensitive) responses to biotrophic pathogens and excessive production to a damaging level during drought stress that can be counteracted to various extents by scavenging enzymes, such as APX, and antioxidant production, such as ascorbate. In addition, ROS can serve in stress signaling, e.g., ROS produced by membrane-bound RBOHs in the apoplast can trigger responses from neighboring cells that may lead to a systemic plant response (Choudhury et al., 2017).

Powdery mildew (PM) *O. neolycopersici* is an important pathogen of tomato and provides a good pathogen-crop model system for studying interactions between abiotic and biotic stress, as various tomato *Ol* genes for PM resistance or susceptibility (*Ol*) are available (Kissoudis et al., 2017; Bai et al., 2018). The partial resistance conferred by *Ol-1* is associated with a slow hypersensitive response (HR) and the underlying gene is most probably not a classical R gene. *Ol-4* on the other hand is a classical R gene, i.e., an NRL gene providing a fast HR. The third gene, *ol-2*, is a mutant of the susceptibility (S) *Mlo* gene and its resistance is associated with papilla formation (Bai et al., 2005). Using tomato lines carrying these various *Ol* resistance genes in the background of tomato cv Moneymaker (MM), we showed previously that resistance to PM was influenced by salt stress intensity and that this depended on the resistance mechanism (Kissoudis et al., 2016). PM resistance conferred by the *Ol-1* gene, but not *ol-2* and *Ol-4*, was partially comprised under mild salt stress (Kissoudis et al., 2016). The differential effect of salinity on the effectiveness of the three *Ol* resistance genes appeared to be linked to ETH and ABA hormonal signaling pathways (Kissoudis et al., 2017). When combined with a mutation leading to ethylene overproduction (*epinastic*), the resistance conferred by *Ol-1* and *ol-2* was considerably more affected under combined salt stress and powdery mildew infection, while the *Ol-4*-conferred resistance remained intact (Kissoudis et al., 2017). When combined with an ABA deficiency mutation (*notabilis*), the *Ol-1*-conferred PM resistance was enhanced, but plant growth was severely affected by salt and combined stress. On the other hand, the *ol-2* line with the ABA deficiency mutation showed lower PM resistance, which was partially restored in combination with salt stress.

Drought is one of the most severe abiotic stress factors for plant productivity. In the present study, we investigated the responses of these tomato lines carrying the *Ol-1*, *ol-2*, and *Ol-4* gene under drought stress, PM infection, and combined drought/PM stresses. Our study aimed to gain insight into the effect of combined abiotic and biotic stresses on tomato, with a focus on (1) the impact of drought stress on powdery mildew infection, (2) the influence of drought on different types of tomato resistance to powdery mildew, and (3) the involvement of underlying signaling pathways by expression analysis of marker genes for SA, JA, ABA, and ETH hormonal pathways, for ROS scavenging, and source-sink relationships.

## Materials and Methods

### Plant and Fungus Materials

The near-isogenic lines (NILs) with the introgressed *Ol*-genes (*Ol-1*, *ol-2*, and *Ol-4)* were used as PM resistant lines Bai et al. (2005). Their background cv. MM was used as the susceptible control. The pathogenic fungus *O. neolycopersici* Wageningen isolate (Bai et al., 2003) was obtained from infected MM plants that were maintained in a greenhouse compartment at 20 ± 3°C with 70 ± 15% relative humidity (RH).

### Experimental Conditions

Two independent experiments were carried out in two different years, in 2015 and 2019, at the Unifarm greenhouse facilities of Wageningen University & Research. In both experiments NIL-*Ol-1*, NIL-*ol-2* and NIL-*Ol-4* were evaluated. For both experiments, the greenhouse air humidity was maintained at 70%, with a photoperiodic regime of 16 h light and 8 h dark. Additional lighting (100 Wm^–2^) was used when the incoming shortwave-radiation was below 200 Wm^–2^. The plants were grown in pots filled with peat medium and watered with 1/2 strength Hoagland’s nutrient solution.

In both experiments 1 and 2, the plants were divided into two groups, one with no powdery mildew inoculation and another inoculated with powdery mildew. The non-inoculated and inoculated plants received the same water limitation treatments.

### Stress Treatments

The first experiment was done with three different levels of water limitation to assess the response of the plants to drought as well as combined powdery mildew and drought. Based on the stress response of the plants in the first experiment, a water limitation treatment that was the most optimal for evaluation of the interaction between the drought and powdery mildew response was chosen for the second experiment.

In the first experiment, three-week-old tomato plants were exposed to three levels of water limitation and watering was given in 2-day intervals. For control conditions, the plants were watered with 400 ml to maintain the soil moisture Θv up to 50%. For the first water deficiency level, termed “Mild drought stress” (D1 and DPM1, without and with powdery mildew infection, respectively), the plants received 200 ml every 2 days to maintain soil moisture Θv range between 22 and 30%. For the “severe drought stress” (D2 and DPM2, without and with powdery mildew infection, respectively) treatment, the plants were watered with 120 ml to maintain the soil at a moisture Θv range between 18 and 20%. For most severe drought treatment, the plants received only 80 ml every 2 days, and this was referred to as ‘Dry down’ (D3 and DPM3, without and with powdery mildew infection, respectively). For each drought treatment, half of the plants were inoculated with tomato PM *O. neolycopersici* by uniformly spraying a suspension of fungal conidia (5 × 10^4^ conidia.ml^–1^) at 8 days after the initiation of drought treatment and grown for another 20 days after inoculation.

The second experiment focused on moderate drought stress. Four-week-old tomato plants with 3–4 fully expanded leaves were watered every day with 400 ml/day to maintain soil moisture Θv up to 50% for the control conditions. The moderate drought stress (D and DM, without and with powdery mildew infection, respectively) was imposed by ceasing the irrigation for 4 days until the moderate drought level (Θv range between 20 and 24%) was reached. Thereafter the plants were re-watered every day with 120–180 ml/day to maintain a soil moisture Θv range between 20 and 24%. Half of the plants in each treatment were sprayed with a suspension of 2 × 10^4^ conidia.ml^–1^ at 6 days after the initiation of the drought treatment and grown for another 20 days after inoculation.

### Plant Phenotyping

Plant phenotyping included stomatal conductance measurements using an SC-1 Leaf Porometer (Decagon Devices, MeterGroup, Germany) and chlorophyll content using a SPAD-502 meter (Konica Minolta Sensing Europe B.V., Netherlands). In the first experiment, stomatal conductance was measured on the fourth or fifth leaf from the bottom and chlorophyll content on the bottom leaves (second leaf from the bottom) and top leaves (7–8th from bottom) at 6 dpi (days post-inoculation). In the second experiment, stomatal conductance and chlorophyll content were measured on middle leaves (5–6th leaves from the bottom) at 3 dpi. Plant height and shoot fresh weight (FW) were measured at the end of each experiment. Stress-induced growth reduction was calculated as the ratio of fresh shoot biomass under stress conditions and under control conditions for each genotype, expressed as a percentage.

### Fungal Pathogen Quantification

For the quantification of relative fungal biomass, plant and fungal genomic DNA (gDNA) was isolated from infected leaves collected at 14 dpi using DNeasy mini kit (Qiagen, Germany) for experiment 1 and at 13 dpi using an adapted CTAB protocol (Doyle and Doyle, 1987) for experiment 2. Relative fungal biomass was quantified by real-time PCR using 20 ng of gDNA as a template for amplification. PCR was performed using primer pair Fw-On-CGCCAAAGACCTAACCAAAA and Rv-On-AGCCAAGAGATCCGTTGTTG, designed on internal transcribed spacer sequence 1 sequence specific to *O. neolycopersici* (GenBank accession number EU047564) (Huibers et al., 2013) and Fw-EF-GGAACTTGAGAAGGAGCCTAAG and Rv-EFCAACACCAACAGCAACAGTCT for tomato reference gene Elongation Factor 1α (Ef1α). Relative fungal biomass was calculated using the 2^–ΔCt^ method (Livak and Schmittgen, 2001) with tomato EF1α as the reference gene.

### cDNA Synthesis and Gene Expression Study

To evaluate the expression of marker genes for stress signaling pathways under different treatments, the third and fourth leaf counting from the bottom were sampled at 7 dpi, when pathogen mycelium growth was not yet visible. RNA for gene expression analyses was isolated with the RNeasy plant mini kit (Qiagen, United States), and the RNA was treated with DNAse I (Invitrogen, United States) to eliminate residual DNA. cDNA synthesis was performed with a 1 μg RNA template using the iScriptTM cDNA Synthesis Kit (BioRAD, United States). qRT-PCR was conducted using the iQ SYBR Green supermix (Bio-Rad, United States) and the CFX96 Real-Time system (Bio-Rad, United States). The reaction mix for two technical replicates contained 11.25 μl iQ SYBR Green super mix,0.68 μl Forward primer (10 μM),0.68 μl Reverse primer (10 μM), and 4.5 μl cDNA (5 ng/μl) template. The final volume for a single reaction was a 10 μl reaction mix. Thermocycling conditions were 95^0^C for 3 min, followed by 40 cycles of 95^0^C for 15 s and 60^0^C for 1 min.

The primers used to monitor the expression of the marker genes are provided in Supplementary Table 1. Relative expression was calculated using the 2^–ΔCt^ method (Livak and Schmittgen, 2001) with tomato EF1α as the reference gene.

### Statistical Analysis

Experiments were arranged in a split-plot design. Statistical analyses were performed using Genstat 19th edition (VSN International Ltd., United Kingdom). Significant differences between the parameters of plants under different treatments were determined by ANOVA *post hoc* tests in conjunction with Fisher’s protected least significant difference test (*P* < 0.05). Significant differences are indicated by different letters in the figures.

## Results

### Plant Responses to Drought Stress (Mild and Severe) and Powdery Mildew Infection

To assess the effect of drought stress on plant growth and fungal development, we first examined tomato plants of the susceptible control MM and the NIL-*Ol*-lines under mild drought, severe drought, PM infection, and combined drought and PM infection treatments.

**FIGURE 1:**
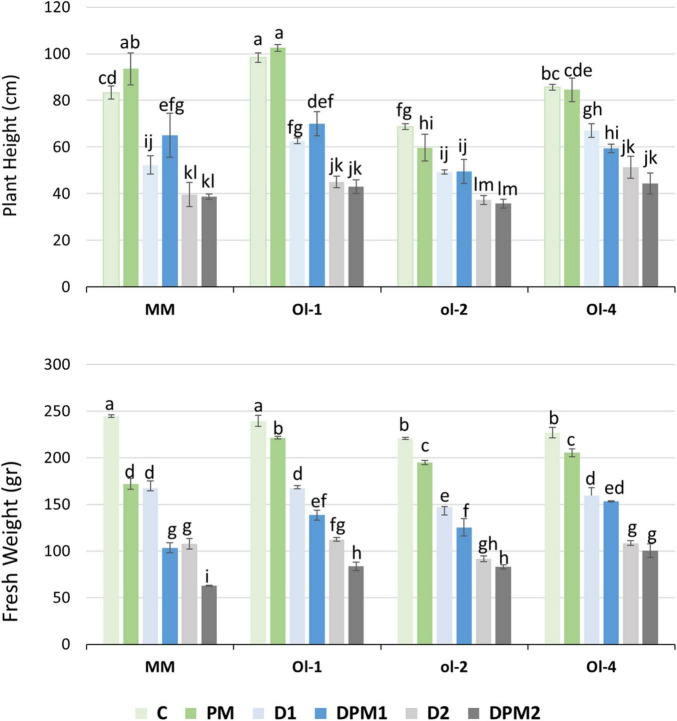
Plant height and shoot fresh weight under control conditions (C), mild drought stress (D1), severe drought stress (D2), powdery mildew (PM) infection (PM), combined D1 and PM (DPM1), and combined D2 and PM (DPM2). Data represent means ± SEM of three biological replicates. Different letters indicate significant differences (*P* < 0.05) compared to Moneymaker (MM) under control conditions.

Under PM infection, plant heights (PH) were slightly reduced only in NIL-*ol-2* (Figure 1, upper panel). While, shoot fresh weights (FW) of all the evaluated genotypes were significantly reduced, with MM showing the highest reduction of all genotypes (32% compared to 7–10% for the NIL lines) (Figure 1, lower panel). Imposition of drought stress reduced PH as well as FW significantly, with severe drought stress resulting in the highest growth penalty with respect to control conditions (Figure 1). FW decreased further under combined drought and PM infection in both MM and NIL-*Ol-1*, with combined severe drought and PM infection leading to the highest reduction. Under mild drought stress, the reduction of FW for MM was 31.5%, while under severe drought stress, the reduction reached 56%. Under combined stress (DPM), MM had the highest SFW reduction under drought treatments of all genotypes (57.7% under combined mild drought and 74% under combined severe drought stress treatment). NIL-*Ol-4* showed a FW reduction of 9.5, 30, and 52% under PM infection, mild drought, and severe drought stress, respectively. Under combined stress, the reduction was the same as under drought stress alone. NIL-*Ol-1* had the least FW reduction under PM infection compared to other plant lines (7.6%), while under mild drought the reduction was 30%. Fresh weight reduction in NIL-*ol-2* was 11.7 and 33% under PM and mild drought, respectively. NIL-*Ol-1* and -*ol-2* had similar FW reductions under severe drought, combined mild drought, and combined severe drought of around 50, 40, and 60%, respectively.

To observe the consequence of infection on stomatal behavior, we measured stomatal conductance under PM treatment. Stomatal conductance under PM was significantly higher (29%) than under control conditions (Supplementary Figure 1). Stomatal conductance decreased under mild drought, severe drought, and their combined stresses compared to control conditions. The reductions under the various stresses ranged from 43 to 53% but were not significantly different between the drought and combined treatments. We did not observe significant differences between genotypes under the treatment conditions, apart from MM and NIL-*Ol-4* under powdery mildew infection. MM and NIL-*Ol-4* showed the highest stomatal conductance compared to other genotypes under all treatment conditions with values of 182 and 167 mmol m^–2^ s^–1^, respectively.

The treatments significantly affected chlorophyll content in the upper leaves (7–8th from bottom, Supplementary Figure 2) but not in the bottom leaves (second leaf from the bottom, Supplementary Figure 3). Chlorophyll content under control conditions was the same as under PM infection. Under mild and severe drought stress and under these stresses combined with PM the chlorophyll content was 10–20% higher than under both control and PM in the upper leaf. There were no significant differences between the drought stress levels. We also did not observe significant differences between genotypes under any of the treatments (Supplementary Figure 2).

### Impact of Mild and Severe Drought Stress on Powdery Mildew Infection

**FIGURE 2:**
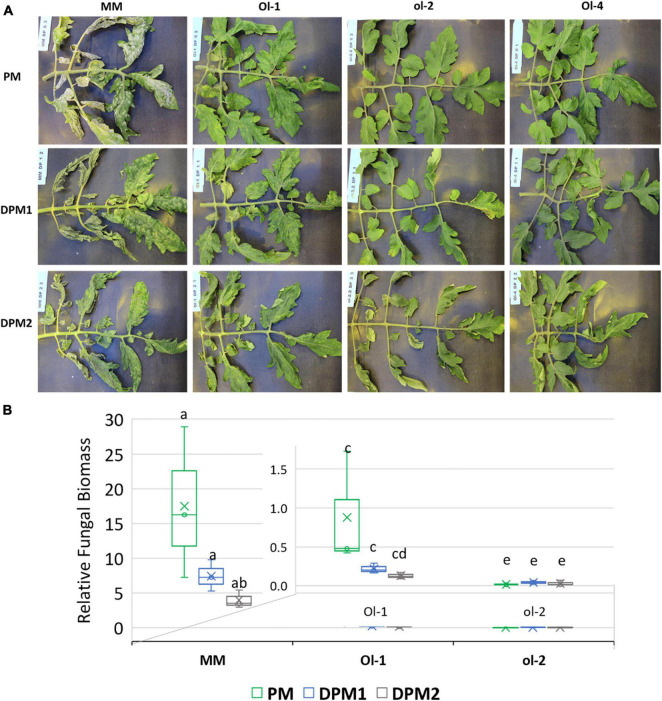
**(A)** Disease symptoms visible as fungal sporulation on the adaxial surface of MM, Ol-1, and Ol-2 leaves under PM and combined stress. **(B)** Relative fungal biomass in MM and Ol lines under powdery mildew infection alone (PM) and in combination with mild (DPM1) and severe (DPM2) drought stress levels. Relative fungal biomass was calculated using the 2^– ΔCt^ method (Livak and Schmittgen, 2001) as the ratio between fungal and tomato gDNA. Leaf samples for fungal biomass quantification and pictures were taken at 14 dpi.

The impact of drought stress on infection levels of PM was assessed by comparing disease development under PM inoculation with that under combined stress. PM infection alone resulted in heavy sporulation on the surface of the leaves of MM (Figure 2A), and this was confirmed by high relative fungal biomass quantification (Figure 2B). Resistant NIL-*Ol-1* also had sporulation on the surface of the leaves, but far less than MM with very low fungal biomass values. Leaves of NIL-*ol-2* and NIL-*Ol-4* did not show any fungal sporulation.

Under combined stresses, mild and severe drought slightly reduced PM infection on susceptible MM and NIL-*Ol-1* (Figure 2A). The fungal biomass on MM and NIL-*Ol-1* slightly decreased under both mild and severe drought stress, with PM combined with severe drought resulting in more reduction compared to PM infection alone (Figure 2B). Disease symptoms on the completely resistant NIL-*ol-2* and NIL-*Ol-4* were not visibly affected by drought stress (Figure 2A).

### Plant Responses to Moderate Drought Stress and Powdery Mildew Infection

Severe drought stress affected PM development in susceptible MM (and NIL-*Ol-1*) slightly more than mild drought, while D3 (dry-down treatment) affected the plants too much to still be able to assess PM infection levels. Therefore, in the second experiment, we chose to focus on a moderate drought regime that allowed us to study the effects of drought on PM infection in more detail and validate the results of the first experiment.

**FIGURE 3:**
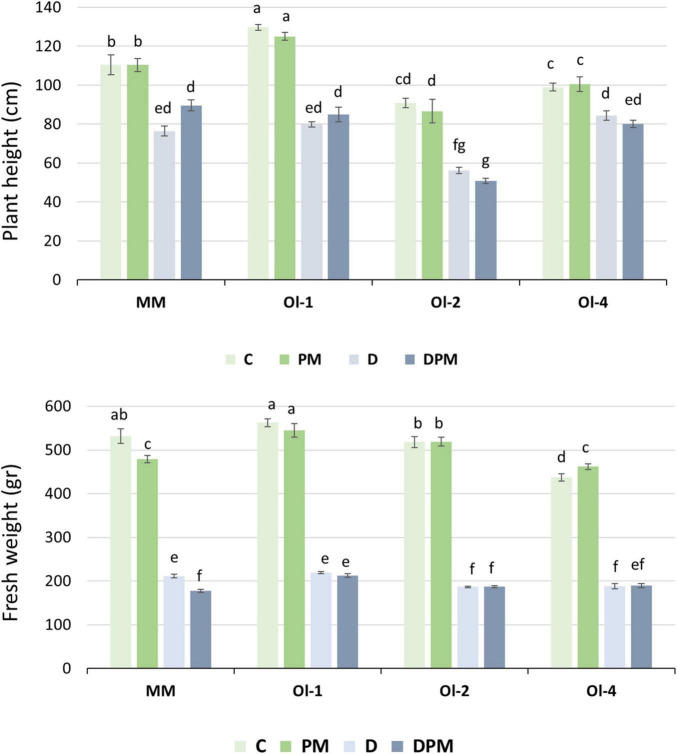
Plant height and shoot fresh weight under control conditions (C), moderate drought stress (D), PM infection (PM), combined D and PM (DPM). Data represents means ± SEM of four biological replicates. Different letters indicate significant differences (*P* < 0.05) compared to MM under control conditions.

In the second experiment, the PH of the genotypes was hardly affected by PM infection but decreased significantly under moderate drought stress (Figure 3). PH of NIL-*ol-2* suffered the highest reduction of all genotypes (44 and 47%, respectively) under drought stress and combined drought stress and PM infection, while NIL-*Ol-4* was the least affected.

Unlike in experiment 1, in which the fresh weight of all genotypes significantly decreased under PM infection, in experiment 2 PM infection did not negatively affect shoot FW of the NIL lines, but it was significantly reduced (10%) in MM. Under moderate drought and combined stress, FW of all genotypes was significantly affected (comparable to experiment 1), with NIL-*Ol-4* showing the least FW reduction (57%). In the NIL lines, FW reduction under combined stress was similar to that under drought stress alone. For MM, the fresh weight reduction was around 60% under drought stress and it was decreased further to 67% under combined stress.

In line with the first experiment, stomatal conductance was reduced under moderate drought stress (69% of that under control conditions) (Supplementary Figure 4). We did not observe significant differences among the genotypes (except for stomatal conductance being significantly lower for NIL-*ol-2* than for the other genotypes under control conditions). Plants under moderate drought stress did not show significant changes in chlorophyll content of the middle leaves compared to control conditions (Supplementary Figure 4). This result was in line with the first experiment with regard to chlorophyll content in the bottom leaves under mild and severe drought stress.

### Moderate Drought Stress Reduces Powdery Mildew Severity in Susceptible Tomato Plants

**FIGURE 4:**
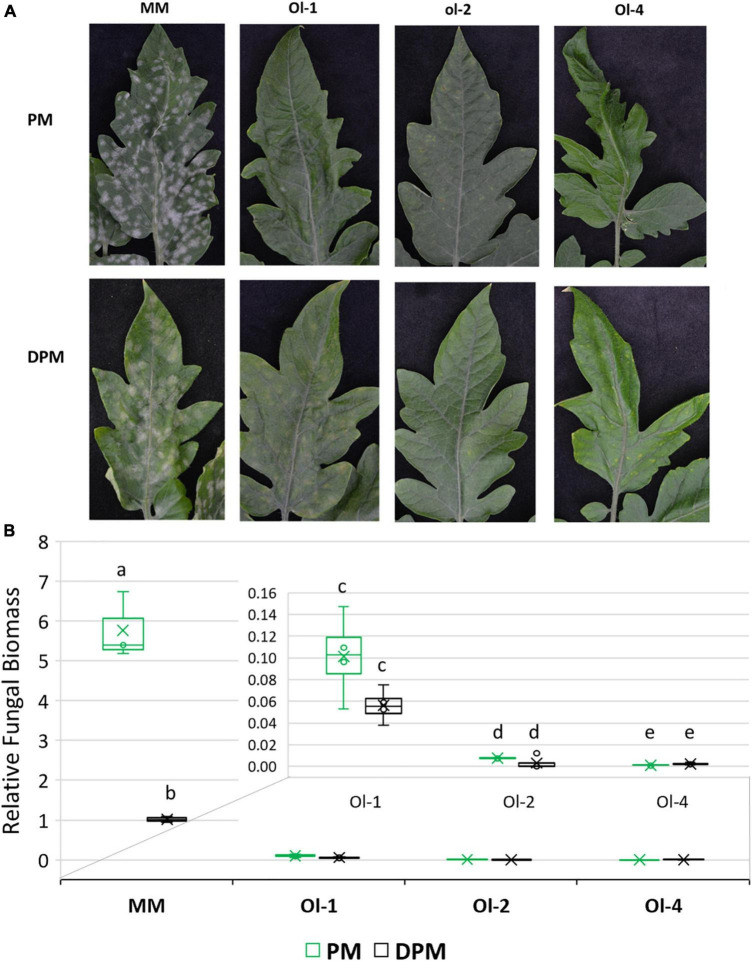
**(A)** Disease symptoms visible as fungal sporulation on the adaxial surface of MM and Ol lines leaves under powdery mildew infection alone (PM) and combined moderate drought and powdery mildew infection (DPM). **(B)** Relative fungal biomass in MM and Ol lines under PM and combined stress DPM. Relative fungal biomass was calculated using the 2^– ΔCt^ method (Livak and Schmittgen, 2001) as the ratio between fungal internal transcribed spacer sequence and tomato EF1α. Leaf samples for fungal biomass quantification and for making pictures were taken at 13 dpi.

On the infected leaves of susceptible control MM plants, heavy PM sporulation was observed under PM treatment (Figure 4A) that was correlated with a high value of fungal biomass (Figure 4B). NIL-*Ol-1* also showed sporulation on the leaves, but far less than MM and also much lower relative fungal biomass. NIL-*ol-2* and NIL-*Ol-4* plants had no fungal sporulation with only a few yellow spots on the leaves and very low fungal biomass, in line with the strong PM resistance conferred by the *ol-2* and *Ol-4* genes.

Under combined drought and PM treatment, disease severity was decreased in MM and NIL-*Ol-1*, whereas in lines NIL-*ol-2* and NIL-*Ol-4* resistance were unaffected. Fungal biomass and fungal sporulation were significantly reduced under drought in MM compared to powdery mildew infection alone (Figures 4A,B). NIL-*Ol-1* showed a slight reduction in fungal biomass under drought. Fungal biomass on lines NIL-*ol-2* and *Ol-4* was already very low and was not affected by drought stress.

Experiments 1 and 2 showed that with increasing drought stress severity, fungal biomass in MM and NIL-*Ol-1* tended to decrease, most significantly in MM. In lines *ol-2* and *Ol-4*, the fungal biomass was consistently very low under all treatments.

### Expression of Genes Involved in Stress Responses Under Individual and Combined Stresses

**FIGURE 5:**
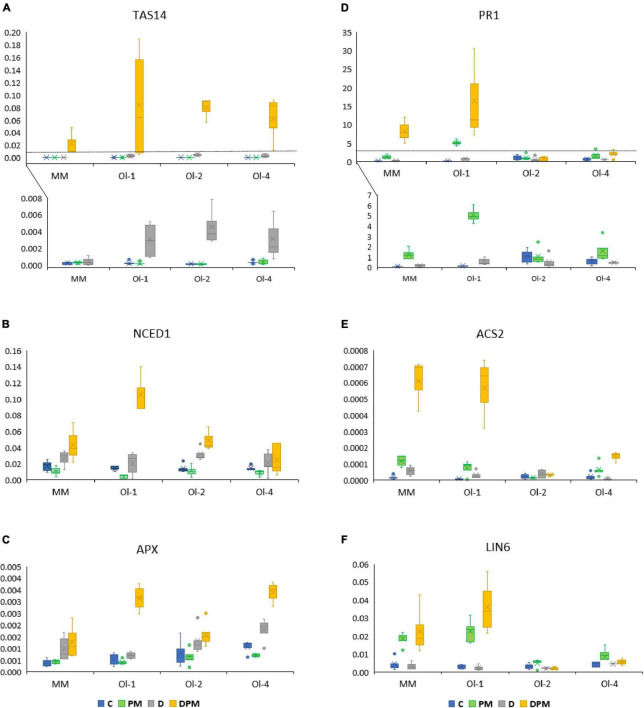
**(A–F)** Expression of tomato marker genes for hormonal, abiotic and biotic stress signaling pathways in leaves of MM, NIL-*Ol-1*, NIL-*ol-2*, and NIL-*Ol-4*, (written as Ol-1, ol-2, and Ol-4 in the figure) relative to the housekeeping gene *SlEF1*α. C is control, PM is powdery mildew infection D is drought stress, DPM is combined drought and powdery mildew infection. Within each treatment, five plants per genotype were used for analysis.

To obtain insight into the molecular mechanisms potentially involved in the response to combined PM and drought stress, we assessed the expression pattern of seven marker genes (listed below) for signaling pathways known to be involved in the drought and pathogen stress response. We analyzed the expression of these genes using treated plants in experiment 1 (Supplementary Figure 6) as well as in experiment 2 (Figure 5). The trends in expression differences were comparable for most genes and stress treatments, therefore we focus below on the more detailed results of the second experiment.

The *SlTAS14* gene encodes a dehydrin, a member of the group 2 late-embryogenesis-abundant (LEA) proteins, and is known to be ABA-responsive and highly induced under osmotic stress. Under control conditions and PM treatment, *SlTAS14* expression was hardly present in all the lines (Figure 5A and Supplementary Figure 6A). However, it was strongly induced under drought stress, and even significantly further induced under combined stress in all tested lines. Compared to MM, the *Ol*-lines showed higher expression levels under drought stress and combined stress relative to control conditions.

The drought stress-responsive *SlNCED1* gene encodes a 9-cis-epoxycarotenoid dioxygenase that catalyzes oxidative cleavage of 9-cis-epoxycarotenoids neoxanthin and violaxanthin to xanthorins, a key step in the biosynthesis of abscisic acid. *SlNCED1* expression was induced under drought stress as well as combined stress in all lines (Figure 5B and Supplementary Figure 6B). NIL-*Ol-1* showed the highest induction levels compared to other genotypes (6.6-fold under combined drought stress and PM). Remarkably, the lines that were infected by PM and showed increased fungal biomass (NIL-*Ol-1* and MM) appeared to have a reduction in SlNCED1 expression under PM alone, but had the highest expression induction under PM combined with drought (Figure 5B),

The *SlAPX1* gene encoding an ascorbate peroxidase is involved in ROS scavenging. *SlAPX1* was upregulated under drought stress and even more so under combined stress (Figure 5C and Supplementary Figure 6C). Under combined stress the highest induction was observed in NIL-*Ol-4*, followed by NIL-*Ol-1*, -*ol-2*, and MM ( 10-, 9-, 5-, and 4-fold, respectively). Under drought stress, the expression of *SlAPX1* was also induced in NIL-*Ol-4* (fivefold), followed by NIL-*ol-2*, MM, and NIL-*Ol-1* (four, five, and twofold, respectively).

The *SlPR1* gene encoding a pathogenesis-related protein is involved in the SA signaling pathway (and SA-dependent systemic acquired resistance). *SlPR1* was highly upregulated under combined stress in MM and NIL-*Ol-1* (11-fold and 33-fold, respectively compared to expression levels of control condition; Figure 5D and Supplementary Figure 6D). This gene was also upregulated in these lines under PM treatments without drought.

The *SlACS2* gene encodes a synthase for the ethylene precursor 1-aminocyclopropane-1-carboxylate. *SlACS2* expression under control conditions was very low, and it was highly induced in MM and NIL-*Ol-1* under combined stress (Figure 5E and Supplementary Figure 6E). In NIL-*Ol-4* the expression level was slightly increased, while in NIL-*ol-2* the expression was not affected by any of the treatments.

The *SlLIN6* gene encoding a cell wall invertase is involved in changes in source-sink relationships in response to wounding and pathogen attack. *SlLIN6* was upregulated in MM and NIL-*Ol-1* under PM infection (four and eightfold respectively compared to control conditions), as well as under combined stress (5- and 12-fold) (Figure 5F and Supplementary Figure 6F). In NIL-*ol-2* and NIL-*Ol-4*, only minor changes were observed in expression levels for SlLIN6.

## Discussion

The response of plants that are exposed to abiotic and biotic stress at the same time is characterized by complex interactions between two living organisms – the plant and the pathogen – and the added dimension of adaptation to abiotic stress (Saijo and Loo, 2020; Zarattini et al., 2021). Designing plant breeding programs to achieve resilience to multiple stress factors is important and research is necessary to increase our understanding of interactions between abiotic and biotic stress responses. In an earlier study, we reported that salt stress affected the response of tomato plants to PM and that the nature of this effect depended on the level of salinity. In MM and NIL-*Ol-1*, the level of PM susceptibility was increased under mildly salt stress while under severe saline conditions it was comparable to control conditions. In contrast, the PM resistance level in NIL*-ol-2* and -*Ol-4* was not affected (Kissoudis et al., 2016). Salinity and drought both cause osmotic stress to the plant, and plant response pathways to salinity and drought partly overlap. In this paper, we studied the response to combined drought and PM infection in tomatoes. Unlike salinity, drought decreased PM infection at all drought levels in both MM and NIL-*Ol-1*, with the strongest decrease under the more severe drought conditions. Similar to salt stress, the resistance conferred by the *ol-2* and *Ol-4* genes was not influenced by drought stress. This suggests that although both abiotic stresses induce osmotic stress, salinity and drought interact differently with the basal defense response to PM in MM and slow HR response to PM associated with the *Ol-1* gene.

Water limitation may affect the pathogen resistance of plants in different ways, depending on the crop, pathogen, and drought scenario. Our results show that drought stress reduced disease symptoms in susceptible tomato cultivar (MM) infected with PM, which is in agreement with the results obtained by Achuo et al. (2006). Similar results were also shown in previous studies on other pathosystems, such as Enright and Cipollini (2011) reporting that increased drought severity appeared to decrease PM susceptibility in garlic mustard, and Ramegowda et al. (2013) showing a similar effect of drought on *Sclerotinia sclerotiorum* (a necrotrophic fungus) and *P. syringae* pv. *tabaci* (a hemibiotrophic bacterial pathogen) infection in *Nicotiana benthamiana*. On the other hand, drought stress was shown to increase the severity of root rot caused by the fungi *Rhizoctonia bataticola* and *Fusarium solani* in chickpea (Sinha et al., 2019), and of the blast fungus *Magnaporthe oryzae* in rice after short periods (3 days) of moderate drought (Bidzinski et al., 2016). Also spider mites, *Tetranychus urticae* and *T. evansi*, were more active on drought-stressed tomato (Ximénez-Embún et al., 2016, 2017).

In the susceptible MM as well as the resistant *Ol*-lines, expression of marker genes *SlTAS14* and *SlNCED1* (for ABA biosynthesis and response respectively) and *SlAPX1* for ETH (*SlAPX1*) was induced under drought stress with a higher induction under combined drought/PM stress (Figures 5A–C). This demonstrates that these signaling pathways react to combined stress factors. Interestingly, the expression level of these marker genes was reduced in MM and NIL-*Ol-1* under salt/PM stress (Figure 7 in Kissoudis et al., 2017), which was coincident with the increased susceptibility to PM in these two genotypes (Kissoudis et al., 2017). With the ABA deficiency mutant *not*, the higher PM susceptibility of NIL-*Ol-1* under combined stress with salt was mitigated, corroborating a role for ABA in salt-induced PM sensitivity (Kissoudis et al., 2017). Additionally, Achuo et al. (2006) reported that the reduced susceptibility to PM under drought was associated with an increase in endogenous ABA levels, further suggesting that higher levels of ABA may be associated with lower PM susceptibility for MM and NIL-*Ol-1*.

Varying responses with respect to susceptibility to pathogens under abiotic stresses like drought could be related to differences in interactions between the underlying signaling pathways. For instance, in the rice – *M. oryzae* (hemibiotrophic) system, higher susceptibility was associated with lower expression of defense marker genes, such as *PR3* (Bidzinski et al., 2016), a gene encoding a chitinase that is induced by ETH and JA. The lower susceptibility of *N. benthamiana* to the hemibiotrophic bacterial pathogen after the drought was associated with higher expression of defense marker genes, *PR5*, and *PDF1.2*. The latter gene was inducible by ETH and JA (Ramegowda et al., 2013), like *PR3* of the rice – *M. oryzae* example. Further evidence for the significance of ETH signaling in the combined stress responses and tolerance comes from a study in a completely different pathosystem, *Arachis spp.* and the root-knot nematode *Meloidogyne arenaria*, in which ethylene signaling genes are uniquely induced under combined nematode and drought stress (Mota et al., 2021). Our results showed that ETH (*SlACS2*) and SA (*SlPR1*) markers were upregulated in MM and NIL-*Ol-1* under combined drought/PM (Figure 5), which was associated with lower susceptibility to the biotroph PM (Figure 4). In a previous study, ETH and SA markers were also upregulated under combined stress with salt, yet PM susceptibility was increased (Kissoudis et al., 2016).

Thus, induction of marker genes for signaling pathways is not necessarily associated with increased resistance to pathogens under combined stresses. The crosstalk among different pathways may be more crucial for the outcome on the level of resistance/susceptibility to a certain pathogen under combined stresses. For instance, ETH could negatively interact with SA leading to disrupted PM resistance in NIL-*Ol-1* as shown by the *epi* mutant (ETH overproducer) (Kissoudis et al., 2017). In addition, ABA works antagonistically to JA and ETH pathways, which is thought to be integrated by ERF genes of which some (e.g., ERF1) bind to both abiotic promoter elements (DRE) and biotic promoter elements (ERE) (Xie et al., 2019). In this way, ABA may affect disease resistance, but it can also improve resistance, amongst others through stimulating callose deposition [e.g., against *Blumeria graminis* f.sp. *hordei* (Asselbergh et al., 2008)]. Higher PM susceptibility under combined salt stress was also associated with a decrease in callose deposition in NIL-*Ol-1* (and NIL-*ol-2*) (Kissoudis et al., 2016).

A negative interaction has also been observed between components of abiotic stress signaling dominated by ABA and defense signaling relayed by SA in *Arabidopsis* (Yasuda et al., 2008). In both this study and our previous study on salinity combined with PM infection, SA marker *PR1* was upregulated in MM and NIL-*Ol-1* under the combined stresses, which was associated with opposite effects on PM susceptibility. SA was also reported to be ineffective in the *O. neolycopersici*-tomato interaction (Achuo et al., 2004). In *Arabidopsis*, the ABA–SA antagonism under abiotic stresses was found to change with leaf age (Berens et al., 2019). These observations indicate the complexity of the interactions between hormonal pathways that may change during plant and tissue development. Moreover, other signaling components or factors related to specific effects of the salt and drought regimes applied could be important to the final outcome of the PM infection.

The wall invertase *SlLIN6* was similarly induced under combined stress in MM and NIL-*Ol-1*. Cell wall invertases are important proteins in source-sink relationships, converting sucrose into the hexoses fructose and UDP-glucose. Increased expression of cell wall invertases and reduced glucose export have been reported in *Phytophthora nicotianae*-infected tobacco leaves (Scharte et al., 2005), and was suggested to support metabolic demand for the pathogen defense response (Proels and Hückelhoven, 2014). Cellular co-silencing of *LIN6* and *LIN8 CWIs* in tomatoes reduced the induction of pathogenesis-related (PR) genes together with pathogenesis symptom development (Kocal et al., 2008), suggesting a direct relationship between the increase in cell wall invertase expression and the increase in *SlPR1* and *SlACS2* expression. Cell wall invertases were also shown to play a role in delaying senescence and drought tolerance (Albacete et al., 2015), while PR-proteins have been shown to be involved in processes like SA-induced senescence and leaf abscission as well (Espinoza et al., 2007; Kim et al., 2015; Borniego et al., 2020), suggesting that these genes may be involved in crosstalk between abiotic stress response and disease symptom development. The high induction of dehydrin *SlTAS14* in MM and NIL-*Ol-1*, observed in concord with the pathogenesis and ethylene response under combined stress is likely an indication of the ABA-dependent stress pathway being induced under drought and further enhanced under combined stress, and may also be indicative of increased ROS and ROS scavenging activity (Halder et al., 2018), which is also supported by the high expression levels of *SlAPX1* under these conditions (Figure 5). Thus, the balance of signaling pathways may be crucial for the resistance/susceptibility to PM in NIL-*Ol-1*.

Interestingly, the resistance conferred by the *ol-2* and *Ol-4* gene was unaffected although induction of some marker genes was observed under combined drought/PM as well as salt/PM stresses (this study and Kissoudis et al., 2016), suggesting that these two genes govern a stable PM resistance under abiotic stresses. The *ol-2* gene belongs to a *mlo* mutant that governs broad-spectrum PM resistance across different plant species (Bai et al., 2008). In barley, several studies showed that drought stress could temporarily compromise *mlo*-resistance, which is however dependent on a few other factors such as soil compaction and genetic background (Newton and Young, 1996; Baker et al., 1998; Kusch and Panstruga, 2017). Under salt stress, PM resistance in NIL-*ol-2* was partially broken in a genetic background with an ABA deficiency mutation (*notabilis*), which is potentially due to the reduction of callose deposition (Kissoudis et al., 2017). Thus, it is worthwhile to test the robustness of *ol-2* resistance in different genetic backgrounds and environmental conditions. The *Ol-4* gene belongs to R genes encoding proteins of the class CC-NB-LRR. The robustness of the resistance governed by the *Ol-4* gene under both salt and drought stress could be a feature of NB-LRR genes but could also be specific for *Ol-4*. However, the trade-off is that the resistance conferred by an R-gene is easily overcome by new races of a pathogen. Thus, it is important to develop a resistance genotype with a broad-resistance spectrum by pyramiding R-genes (Kim et al., 2021). Although the resistance conferred by the *ol-2* and *Ol-4* remained unaffected under salt/drought stress, plants suffered growth penalties under these abiotic stress conditions (Kissoudis et al., 2016 and this study). In order to keep the growth penalty resulting from abiotic stress low, it is recommended to combine R-genes with QTLs conferring abiotic stress tolerance, as exemplified by the pyramiding approach to combine drought and blast resistance in rice (Balija et al., 2021).

## Data Availability Statement

The datasets presented in this study can be found in online repositories. The names of the repository/repositories and accession number(s) can be found in the article/Supplementary Material.

## Author Contributions

SS and CK participated in the design of the study, and performed the greenhouse experiment, phenotypic characterization, gene expression analyses, data analysis, and manuscript writing. YV performed the greenhouse experiment, fungal biomass analysis, and gene expression analyses. HV assisted in the greenhouse experiment and gene expression analyses. CVL, GVW, RV, and YB designed the study, supervised the work, and assisted with editing the manuscript. All authors read and approved the final manuscript.

## Conflict of Interest

The authors declare that the research was conducted in the absence of any commercial or financial relationships that could be construed as a potential conflict of interest.

## Publisher’s Note

All claims expressed in this article are solely those of the authors and do not necessarily represent those of their affiliated organizations, or those of the publisher, the editors and the reviewers. Any product that may be evaluated in this article, or claim that may be made by its manufacturer, is not guaranteed or endorsed by the publisher.
