# Supplementary material for: Drought Stress Interacts With Powdery Mildew Infection in Tomato
Source: Front Plant Sci. 2022 Mar 8;13:845379. doi: 10.3389/fpls.2022.845379 (PMC8958004; doi:10.3389/fpls.2022.845379)
Supplement: Supplementary file 1 [file Data_Sheet_1.docx]

Supplementary Table 1. Primers used for expression analyses with qRT-PCR

| **Gene name** |  | **Sequence (5'-3')** | **Gene function** |
| --- | --- | --- | --- |
| *SlNCED1* | NCED1_F | TCGAAAACCCGGATGAACAAGTGA | ABA biosynthesis |
|  | NCED1_R | AACCAGAAACTTTTGGCCATGGTTC |  |
|  |  |  |  |
| *Sl_TAS14* | DHN_TAS_F | CACCATGAGGGGCAACAGCA | ABA responsive dehydrin |
|  | DHN_TAS_R | TCACCTTCATGTTGTCCAGGCATC |  |
|  |  |  |  |
| SlACS2 | ACS2_ F | CGCGATGAGGTTAGGTAAAAGGCA | Ethylene biosynthesis |
|  | ACS2_R | GTCGATTCCCTTAAAAGTGGACGCA |  |
|  |  |  |  |
| *SlLOXD* | LOXD_F | GCAGTACCGGACGCAACACA | Jasmonic acid biosynthesis/response |
|  | LOXD_R | CTGCAAACTTGGGCCGAGGA |  |
|  |  |  |  |
| *SlPR1* | PR1_F | TGGTGACTTCACGGGGAGGG | Salicylic acid/defense response |
|  | PR1_R | CGGACTGAGTTGCGCCAGAC |  |
|  |  |  |  |
| *Sl*APX1 | SlAPX1_F | CCATTTGGAACAATCAGGCACCCG | Antioxidant defence-Redox regulation |
|  | SlAPX1_R | CGGGGCCTCCCGTAACTTCA |  |
|  |  |  |  |
| *SlLIN6* | LIN_F | TTGGTTCAATGGCCTGTTCAAG | Cell wall invertase |
|  | LIN_R | TTCAACGTCAGCCTGTGCAA |  |

Supplementary Figure 1. Stomatal conductance under control conditions (C), mild drought stress (D1), moderate drought stress (D2), PM infection (PM), combined D1 and PM (DP1), and combined D2 and PM (DP2). Data represent means ± SEM of three biological replicates. Different letters indicate significant differences (P<0.05) compared to MM under control conditions.

Supplementary Figure 2 Chlorophyll content of upper leaf under control conditions (C), mild drought stress (D1), severe drought stress (D2), PM infection (PM), combined D1 and PM (DP1), and combined D2 and PM (DP2). Data represent means ± SEM of three biological replicates. Different letters indicate significant difference (P<0.05) compared to MM under control conditions.

Supplementary Figure 3. Chlorophyll content of bottom leaf under control conditions (C), mild drought stress (D1), severe drought stress (D2), PM infection (PM), combined D1 and PM (DP1), and combined D2 and PM (DP2). Data represent means ± SEM of three biological replicates.


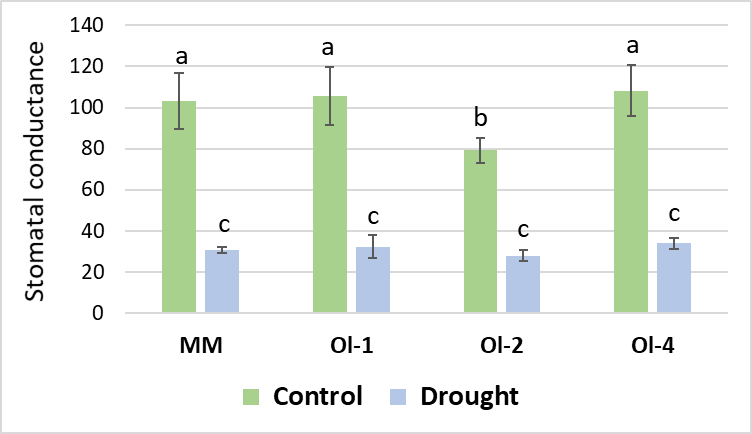

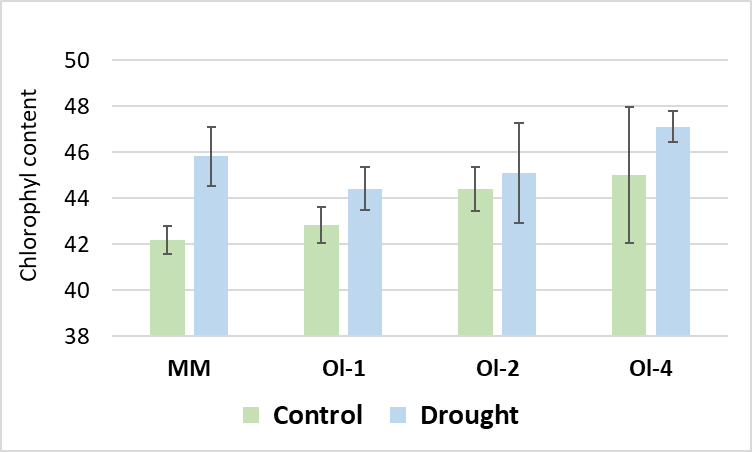


Supplementary Figure 4. A) Stomatal conductance and B) Chlorophyl content of the genotypes under control conditions and moderate drought stress. Data represents means ± SEM of four biological replicates. Different letters indicate significance difference (P<0.05) compare to MM under control condition.

Supplementary Figure 5. Stomatal conductance under control condition (C), powdery mildew infection (PM), mild drought (D1), moderate drought (D2), severe drought (D3), combined mild and PM (DP1), and combined moderate and PM(DP2). Data represents means ± SEM of at least three biological replicates. Different letters indicate significance difference (P<0.05) compare to control condition.

Supplementary Figure 6: Expression of tomato marker genes for hormonal, abiotic and biotic stress signalling pathways in leaves of MM, NIL-*Ol-1*, NIL-*ol-2*, and NIL-*Ol-4*, expressed relative to the housekeeping gene SlEF1α. C=control, M= Mild Drought, S=Severe Drought. With or without Powdery mildew treatment is indicated with + and -, respectively.
